# Selection of Low‐Density EEG Montages for Optimal Seizure Detection Performance

**DOI:** 10.1049/htl2.70104

**Published:** 2026-10-26

**Authors:** Shima Abdullateef, Iris Soare‐Nguyen, Brian Jordan, Ailsa McLellan, Vera Nenadovic, Javier Escudero, Milly Lo

**Affiliations:** ^1^ Usher Institute College of Medicine and Veterinary Medicine University of Edinburgh Edinburgh UK; ^2^ Institute for Imaging, Data and Communications School of Engineering University of Edinburgh Edinburgh UK; ^3^ Combined Paediatric Neuroscience Service Royal Hospital for Children and Young People Edinburgh UK; ^4^ BrainsView Inc. Ontario Canada; ^5^ Muir Maxwell Epilepsy Centre University of Edinburgh Edinburgh UK; ^6^ Paediatric Critical Care Unit Royal Hospital for Children and Young People Edinburgh UK

**Keywords:** channel selection, EEG, epilepsy, low density montage, machine learning

## Abstract

Multi‐channel electroencephalography (EEG) is widely recognised for its ability to non‐invasively capture dynamic brain activity with high temporal resolution. However, conventional EEG systems require meticulous setup, expert interpretation and specialised infrastructure, which limits their applicability in real‐time, resource‐constrained environments such as critical care and emergency settings. As wearable and point‐of‐care EEG technologies gain traction, there is increasing demand for low‐density, automated seizure detection methods that are clinically reliable and scalable. This study presents a data‐driven pipeline for an EEG‐based task, seizure detection, that identifies compact and clinically relevant four‐channel electrode configurations. Using standard 10–20 EEG recordings, the pipeline integrates clinical domain knowledge, preprocessing, feature extraction, and ensemble boosted classification to evaluate seizure detection performance across multiple electrode combinations. The approach was validated on both a heterogeneous local paediatric EEG dataset and an open‐source adult dataset. Our results show that several four‐channel montages achieve robust patient‐independent performance, with central and temporal electrodes frequently appearing in the top‐performing configurations. These findings support the feasibility of accurate seizure detection using low‐density EEG, even across diverse clinical populations. The primary contribution of this work is a generalisable pipeline for selecting compact EEG montages, which balances domain insight and data‐driven optimisation. This framework can be readily adapted to other EEG‐based tasks and offers a practical path toward real‐time wearable EEG monitoring in resource‐constrained environments.

## Graphical abstract

This study presents a data‐driven pipeline for EEG‐based seizure detection, designed to identify compact and clinically meaningful four‐channel electrode configurations. Leveraging standard 10–20 EEG recordings, the pipeline integrates clinical domain knowledge, signal preprocessing, feature extraction and an ensemble boosted tree classifier to evaluate seizure detection performance across a range of electrode combinations. While seizure detection serves as the primary application in this study, the pipeline is generalisable and can be adapted for other EEG‐based classification tasks such as sleep staging, cognitive workload assessment, or brain–computer interface development.

## 1 Introduction

Electroencephalography (EEG) is widely recognised for its ability to non‐invasively capture dynamic brain activity with high temporal resolution [1]. However, the high dimensionality of multichannel EEG recordings introduces significant challenges for EEG‐based tasks, including increased computational burden, risk of overfitting and noise, and difficulties in real‐time deployment. To address these challenges, data‐driven pipelines have been developed to identify minimal yet maximally informative subsets of EEG channels [1]. These methods aim to retain or enhance classification accuracy while reducing the processing demands associated with redundant or noisy signals. They typically integrate robust preprocessing, feature extraction, advanced channel selection techniques and classifier architectures optimised for applications such as brain–computer interface [2], motor imagery [3], seizure detection [4] and emotion recognition [5].

Among the various applications of EEG, seizure detection stands to benefit significantly from real‐time computational analysis, offering clinical value in acute and critical care environments where timely intervention is essential. Of note, electrographic seizures occur frequently in critical and acute care settings. These seizures are subclinical, occurring without any clear clinical markers [6, 7]. Delays in detecting and treating these seizures can worsen neurological outcomes and prolong patients' stays in healthcare facilities [8, 9].

The current gold standard for seizure detection is continuous, multichannel EEG recordings, typically using a minimum of 16 channels combined with video monitoring [10]. This method requires meticulous electrode placement involving specific scalp preparation and precise measurements to ensure accuracy. Moreover, expert neurologists must interpret the prolonged recordings to accurately identify seizure onsets.

However, this practice has several drawbacks. First, scalp preparation and electrode placement are time‐consuming procedures that can only be performed by certified EEG technologists. Second, reviewing EEG recordings to detect seizures is labour‐intensive, requiring specialists to sift through hours of data to identify short seizure events. Third, providing 24/7 access to highly trained clinicians is unsustainable in many clinical settings [11, 12].

To address these challenges, clinicians and researchers have explored the concept of limited montages, which involve selecting a smaller subset of electrodes to optimise the capture of relevant neural activity [13, 14, 15, 16, 17]. Concurrently, advances in wearable EEG technology and artificial intelligence (AI) have driven research into using machine learning models with low‐density montages for automated seizure detection.

The initial stage of most EEG‐based classification pipelines typically involves rigorous preprocessing to improve signal quality by removing noise and artefacts. Raw EEG signals are usually subjected to bandpass filtering to isolate relevant frequency bands such as delta, theta, alpha, beta and gamma. Subsequently, feature engineering techniques are applied to robustly characterise seizure‐related patterns in EEG signals. Commonly used features include entropy [18, 19, 20], phase synchrony [21], root‐mean‐square [22] and line length [23, 24], also known as coastline, among other linear and nonlinear metrics. These features serve as input to machine learning models trained to perform a classification, distinguishing, for example, between seizure and non‐seizure EEG activity. For a comprehensive overview of state‐of‐the‐art seizure detection approaches, readers are referred to [25, 26].

Research in automated seizure detection generally falls into two categories: patient‐specific and patient‐independent approaches. Patient‐specific models are trained and evaluated using data from the same individual, allowing the model to learn individual‐specific seizure characteristics [20, 27, 28, 29, 30, 31]. In contrast, patient‐independent models are developed to detect seizures across a diverse cohort of patients, with the aim of achieving generalisability across subjects [32, 33, 34, 35].

In patient‐specific frameworks, channel selection is often personalised based on the individual's seizure morphology and spatial localisation. These approaches leverage data‐driven methods to identify a minimal subset of electrodes that best capture ictal activity for a given patient. For example, a seizure detection system may be configured using only two electrodes strategically selected to monitor the most informative scalp regions [31, 36, 37].

Although patient‐specific models tend to achieve higher accuracy and can be tailored to individuals’ seizure types and corresponding EEG configurations, they pose challenges in critical care environments where multiple patients are monitored simultaneously. In such settings, patient‐independent methods are preferable despite sometimes lower accuracy, as they offer greater scalability and generalisability [34, 35].

Notable examples include the work by Frankel et al. [38], who evaluated a wearable EEG device using 10 channels for automatic seizure detection in adults. Their machine learning model achieved a mean accuracy of 90% compared with expert clinical reviews across subjects. Similarly, Abdullateef et al. [39] investigated automated seizure detection in a paediatric cohort using two electrode configurations: one with eight circumferential and another with four central electrodes. The achieved accuracies of 77.2% and 76.5%, respectively, demonstrated the feasibility of low‐density, patient‐independent seizure detection.

Building on our previous work [39], which demonstrated the feasibility of patient‐independent seizure detection using low‐density EEG, this study introduces a generalisable and data‐driven pipeline for EEG channel subset selection in classification tasks. While seizure detection serves as the primary case of use due to its high clinical relevance and well‐annotated datasets, the core objective is to develop and evaluate a robust framework capable of identifying compact yet informative channel configurations suitable for a range of EEG‐based applications.

The proposed pipeline leverages standard 10/20 EEG recordings and integrates channel selection, preprocessing, feature extraction and classification components. We validate the pipeline using two datasets: one local paediatric cohort and one publicly available adult dataset, employing a consistent four‐channel montage structure. To our knowledge, this is the first study to apply a mixed‐method data‐driven analysis to systematically evaluate seizure detection using four‐channel interpatient configurations across age groups and seizure types.

Although we use machine learning methods to classify EEG changes associated with seizures, our primary aim is not to optimise seizure detection accuracy or to introduce a novel classification algorithm, but to demonstrate that detection performance can be maintained using a smaller subset of channels across different cohorts. Our findings demonstrate that four‐channel montages with central and temporal electrodes can effectively detect seizures regardless of their origin, type, or the patient's age group, underscoring the potential of the proposed pipeline to support real‐world, resource‐constrained EEG applications.

The Methods section describes the process for selecting optimal four‐channel combinations and the datasets. This is followed by the Results, which present the performance of the selected montages. Finally, the Discussion interprets the findings in a clinical context, and the paper concludes with key takeaways and future directions.

## 2 Methods

In a mixed‐method data‐informatics study, our approach extends beyond technical or algorithmic development and focuses on translational relevance. Instead of solely relying on technical and often inexplicable computational approaches, we integrated insights from multiple domains, such as clinical expertise and end‐user considerations, into a data‐driven pipeline. Hence, decisions such as channel selection and model evaluation were guided not only by performance metrics, but also by practical constraints and domain knowledge. We developed a pipeline to select a predefined set of four‐channel montages for patient‐independent seizure detection using local and open‐source datasets, with the aim of facilitating future adoption in real‐world clinical settings.

### 2.1 Channel Selection Pipeline

Based on clinical expertise and prior literature [40], a subset of channels with high clinical relevance for seizure detection was identified and designated as Collection 1. This set included electrodes C3, C4, T3, T4, O1, O2, P3, and P4. To construct Collection 2, additional electrodes were selected from the remaining positions in the standard 10/20 system, as illustrated in Figure 1. Notably, electrodes were selected in pairs to ensure coverage across both hemispheres and to account for the diverse localisation of seizure onset. This process yielded a total of 21 distinct four‐channel configurations (Table A1).

**FIGURE 1:**
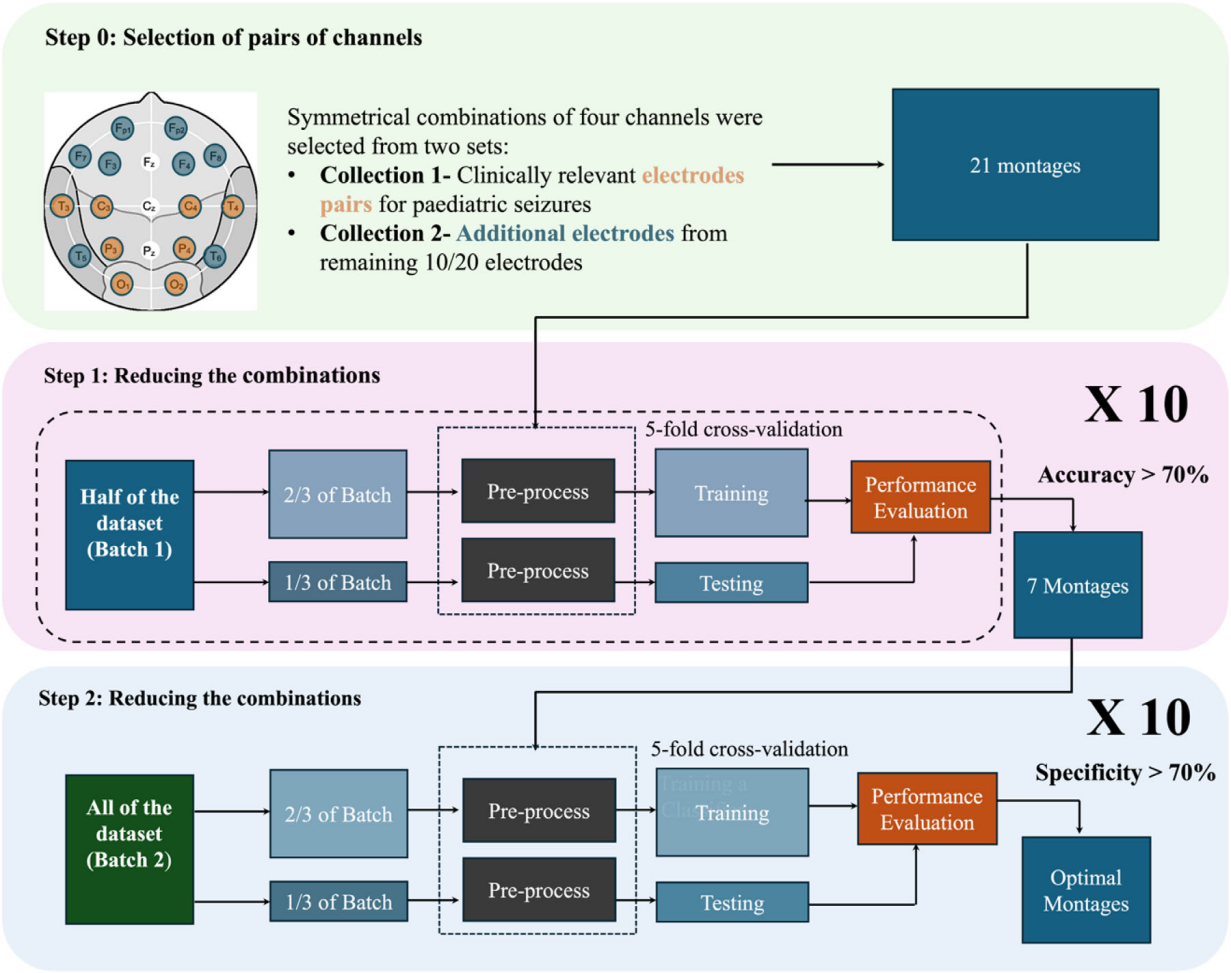
Schematic representation of the proposed pipeline for selecting predefined electrode combinations for seizure detection.

As illustrated in Figure 1, following the selection of the 4‐channel configurations (Step 0), half of the subjects from each cohort (*N*_siena_ = 7; *N*_local_ = 20) were reserved for the initial analysis. The input data was divided into three subsets: training, validation and testing. The training set, comprising the majority of the data, was used to fit an ensemble boosted tree classifier. A validation set, within the training set, was used to tune model parameters. The testing set was used to assess generalisation to unseen data. Specifically, two‐thirds of the available data were used for training, and the remaining third was allocated for testing.

To further evaluate the robustness of the model, five‐fold cross‐validation was performed, with four folds used for training and one for validation. This entire process was repeated ten times using bootstrapping to estimate the variability in classifier performance [41], with different subjects drawn for the training and test set in each iteration.

### 2.2 Channel Preparation

The selected channels were pre‐processed using a bandpass filter with a cut‐off frequency of 0.75—4 Hz, targeting the delta band. The bandpass filter was implemented using zero‐phase digital filtering (order = 80) in MATLAB 2023b. To ensure consistency across different recordings, all signals were re‐sampled to 200 Hz. No additional artefact removal steps were implemented to preserve the authenticity of the recordings, including the typical noises present in a clinical environment. Phase amplitude coherence and phase synchrony were extracted to highlight the changes in the EEG signal during seizures. For detailed information about the feature extraction, readers are referred to [39].

### 2.3 Classification

In this binary classification task, the classifier distinguishes between seizure and background activity. To evaluate classifier performance, we used clinician‐provided annotations marking seizure onsets as the gold standard. The model's effectiveness was assessed using statistical measures such as specificity and accuracy for both validation and test comparisons. The classifier used was an ensemble of decision trees, specifically boosted trees trained using the AdaBoostM1 method, with a maximum of 20 splits per tree.

Classification performance was evaluated using four standard classification metrics: accuracy, sensitivity, specificity and precision. In Step 1, montages achieving median accuracy of at least 70 percent are selected for further evaluation.

In Step 2, the analysis was extended to include all of the subjects from each cohort, including those used in Step 1 (*N*_siena_ = 14; *N*_local_ = 39). Only the montages with high median accuracy identified in Step 1 were selected for further evaluation in this step of the pipeline. For each subject, seven montages were used as input to train an ensemble boosted tree classifier. For each subject, seven montages were used as input to train an ensemble boosted tree classifier. All subjects included in Step 1 were retained in the training set. The remaining subjects were randomly split so that, overall, two‐thirds of the full dataset were used for training and the remaining one‐third were reserved for testing.

As in the previous step, five‐fold cross‐validation was conducted within the training set, using four folds for training and one fold for validation to tune model parameters. The bootstrapping process was repeated ten times to estimate variability in classifier performance. Specificity was again chosen as the selection metric for this step, as a primary concern in monitoring devices is the high rate of false alarms and the consequent ‘alarm fatigue’ among critical care bedside staff [42].

### 2.4 Datasets

#### 2.4.1 Siena Dataset

This open‐access dataset [43] was acquired at the Unit of Neurology and Neurophysiology of the University of Siena. It contains EEG recordings from 14 patients, with a total of 47 seizures. The subjects include nine males and five females, with ages between 20 and 71. The recordings consist of 29 or 31 EEG electrodes, whose placement followed the 10/20 international system standard. The sampling frequency is 512 Hz, with a unipolar reference. The data are reviewed and annotated by an expert clinician, and they contain: 13 patients with temporal epilepsy and 1 with focal epilepsy; this also translates to 13 patients having lateral localisation (4 in the right hemisphere and 9 in the left hemisphere) and 1 having bilateral localisation.

#### 2.4.2 Local Single‐Centre Dataset

Forty fully anonymised and routinely collected recordings were retrospectively obtained from a single‐centre and subsequently analysed offline (NHS Lothian R&D No. 2021/0021). Prior consent for the analysis of retrospective EEG data was acquired from the parents or caregivers of the patients. Adherence to all pertinent regulations outlined in the Declaration of Helsinki and data protection protocols was ensured. The data were extracted from the Royal Hospital for Sick Children, Edinburgh, and converted to European Data Format (EDF). Electrode placement followed the 10/20 international system, and a monopolar electrode montage was utilised. Subjects were chosen randomly, with the inclusion criteria of having at least one seizure, thereby encompassing various seizure types and recording frequencies. During routine EEG collection, a neurologist documented all paroxysmal events along with their descriptions and seizure onsets. As the common recording reference, the electrode Cz’ was used. Our cohort included patients with various aetiologies and had a mean age of 9.3 ± 5 years (mean ± STD). One subject was excluded, as it had no seizures.

## 3 Results

This section presents the results of the proposed data‐driven channel selection pipeline on the two datasets described earlier. Our proposed pipeline aims to identify the most informative EEG channels from the 10–20 system, with the objective of reducing the number of electrodes while maintaining seizure detection performance across diverse patient groups and seizure types.

### Siena Adult Dataset

Different four‐channel montages were evaluated using half of the Siena dataset (*N* = 7). An ensemble boosted tree classifier was trained and tested on each montage, with performance assessed using ten rounds of bootstrapping to ensure robustness. Figure 2 presents the bootstrapped classification results for all 21 montages, ranked by median accuracy. The most accurate configurations are displayed in the top row.

**FIGURE 2:**
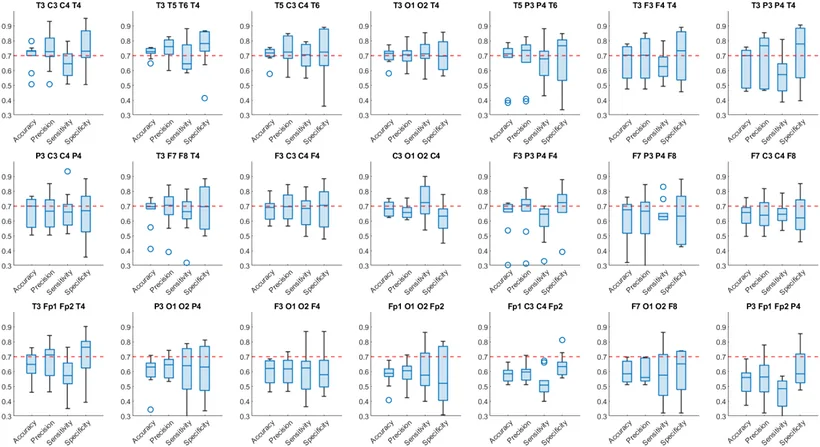
Boxplot representation of Siena dataset showing accuracy, sensitivity, specificity and precision (Step 1). The figures are organised in descending order of median accuracy values, with the first row having the highest median accuracy.

The top seven configurations were then evaluated on the full Siena dataset (*N* = 14) using the same classifier and bootstrapping procedure. It is important to note that each subject in the dataset has a different seizure localisation, which supports the generalisability of the selected montages. The outcome of this step is depicted in Figure 3.

**FIGURE 3:**
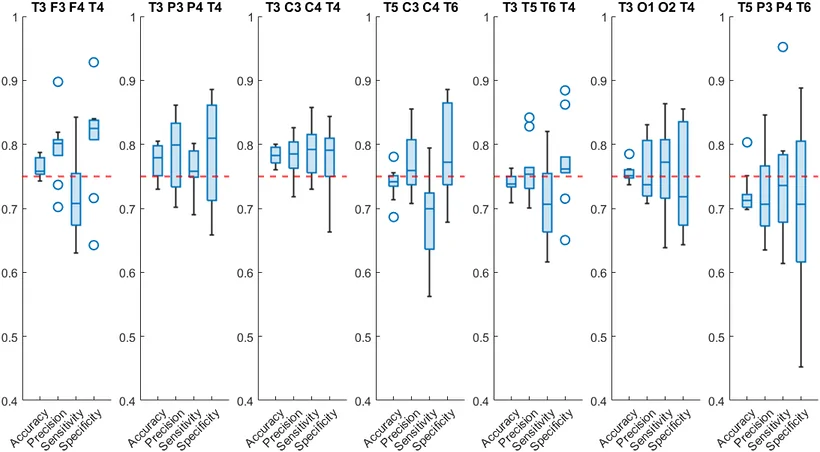
Boxplot representation of the performance of the selected montages on the complete Siena dataset using ten‐fold bootstrapping (Step 2). Montages are organised by their median specificity values, from highest (left) to lowest (right).

### Local Pediatric Dataset

The proposed approach was first applied to a local paediatric EEG dataset using a subset of the data (*N* = 20) and 21 different channel montages. Figure 4 illustrates the bootstrapping performance of ensemble boosted trees, with the montages sorted by median accuracy. The highest‐performing combinations are presented in the top row, highlighting the most effective electrode configurations for seizure detection.

**FIGURE 4:**
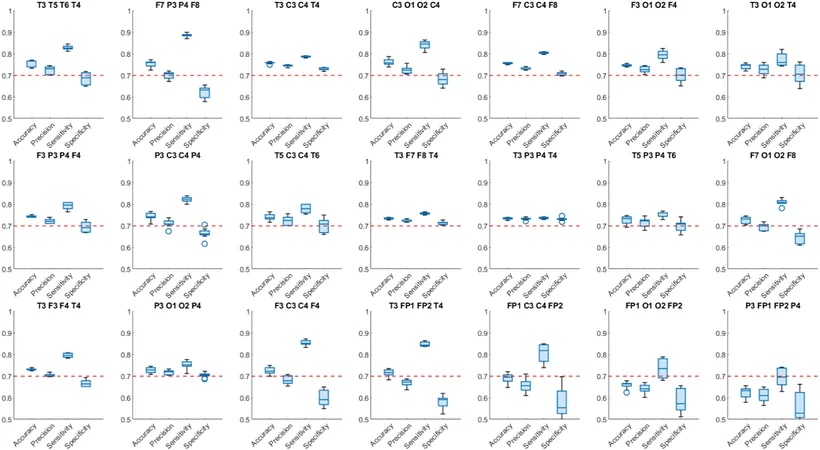
Boxplot representation of the local paediatric dataset showing accuracy, sensitivity, specificity and precision using ten‐fold bootstrapping (Step 1). The figures are organised in descending order of median accuracy values, with the first row having the highest mean accuracy.

Subsequently, the top seven montages were trained on two‐thirds of all subjects and tested on the remaining unseen subjects. All subjects used in Step 1 were retained in the training set, while the newly introduced subjects were randomly split into training and testing subsets. This procedure was repeated ten times, with different random splits of the new subjects in each iteration. The models were evaluated on the full dataset comprising 39 patients, and the resulting performance on the test sets is summarised using boxplots in Figure 5.

**FIGURE 5:**
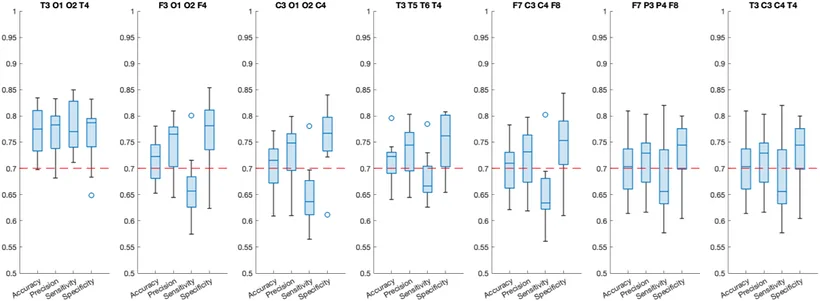
Boxplot representation of the performance of the selected montages in the complete local dataset using ten‐fold bootstrapping (Step 2). Montages are organised by median specificity values, from highest (left) to lowest (right).

### Selected Channels

Table 1 presents the montages that achieved the highest performance across both datasets, which included seizures of varying origins and types. Notably, three of the top seven montages were common to both age groups, suggesting some degree of generalisability. In the paediatric dataset, four‐channel montages with frontal lobe coverage were more prominent, whereas in the adult cohort, montages including central electrodes were more frequently associated with higher classification accuracy. In both datasets, four‐channel configurations with temporal electrodes were the most prominent.

**TABLE 1:** A summary of the top‐performing montages for both datasets. Montages common to both datasets are marked with ‐, * and +.

Rank	(Adult, *N* = 14)	(Paediatric, *N* = 39)
1	T3 F3 F4 T4	**T3 O1 O2 T4^−^**
2	T3 P3 P4 T4	F3 O1 O2 F4
3	**T3 C3 C4 T4^+^**	C3 O1 O2 C4
4	T5 C3 C4 T6	T3 T5 T6 T4^*^
5	T3 T5 T6 T4^*^	F7 C3 C4 F8
6	**T3 O1 O2 T4^−^**	F7 P3 P4 F8
7	T5 P3 P4 T6	**T3 C3 C4 T4^+^**

## 4 Discussions

This study aimed to develop a systematic approach for selecting low‐density EEG channel subsets from the standard 10–20 electrode placement system. The objective was to reduce the number of electrodes required while preserving the classification performance. The proposed method was designed to be generalisable across different EEG‐based classification tasks. In this work, seizure detection was used as the target application to evaluate the performance and clinical unitality of the selected channel configurations. Using a mixed‐method data‐driven approach, we applied the proposed channel selection pipeline to hospital‐acquired EEG recordings and evaluated its utility in patient‐independent seizure detection. The results demonstrate that a subset of as few as four electrodes can achieve over 70% accuracy and over 70% specificity across two datasets representing different age groups. Notably, three‐channel montages consistently appeared in both datasets, suggesting the potential generalisability of the selected configurations. These findings support the feasibility of implementing more efficient and wearable EEG monitoring systems, particularly in long‐term or at‐home settings.

Reducing the number of EEG electrodes can simplify the setup process and decrease the time and effort required for electrode placement, which is particularly beneficial in clinical and ambulatory settings. However, this reduction in spatial resolution may lead to the loss of critical information necessary for accurate diagnosis and seizure detection. Therefore, achieving an optimal balance between signal quality and electrode count is crucial. To address this challenge, we employed an algorithm that integrates key features of EEG signals and achieves accurate seizure detection using only four channels, as demonstrated in previous work [39].

To ensure consistent detection of both focal and generalised seizures, we selected symmetrical unipolar EEG electrode pairs when forming the four‐channel configurations. This approach provides bilateral coverage and increases the likelihood of capturing seizure activity regardless of its localisation. As this study introduces a novel mixed‐methods pipeline that repurposes standard 10/20 EEG recordings for evaluating point‐of‐care‐compatible montages, there are currently no directly comparable methodologies in the literature. Nevertheless, several commercially available point‐of‐care EEG systems, such as Ceribell, Starstim 8 and Epitel [38, 44, 45], also employ symmetrical electrode arrangements, aligning with our design choices and supporting the translational relevance of our findings [10].

Temporal and central electrodes were consistently present in most top‐performing montages identified through our feature selection process. This finding is likely due to the high prevalence of temporal lobe seizures in both adult and paediatric epilepsy populations [46]. Supporting this, 13 out of 14 seizures in the Siena dataset were localised to the temporal region [47]. Central electrodes, meanwhile, may contribute to performance due to their anatomical location. They are positioned near both the temporal and frontal regions, allowing them to capture seizure propagation patterns across multiple cortical areas due to volume conduction [48].

One of the key differences between standard 10–20 EEG recordings and emerging wearable systems such as the Sensor Dot [49] lies in the referencing method. In most hospital‐acquired EEG datasets, recordings are unipolar, where each electrode's activity is referenced to a common point, such as Cz, the earlobe or the mastoid [50]. In contrast, bipolar recordings measure the voltage difference between pairs of adjacent electrodes, which helps reduce the influence of volume conduction and improve spatial resolution [51].

While this study introduces a pipeline for selecting a fixed subset of EEG channels optimised for seizure detection, further exploration of alternative EEG features and classification algorithms is warranted. In particular, deep learning methods have demonstrated strong potential for detecting seizure onsets without the need for extensive preprocessing or handcrafted feature extraction [52]. Future studies could investigate whether the current channel selection pipeline remains effective when paired with end‐to‐end deep learning models, potentially improving both performance and generalisability.

It is important to acknowledge that the classification performance reported in this study may not be competitive with the highest‐performing seizure detection algorithms in the literature. However, our primary focus was not on maximising classification accuracy. Instead, we focused on developing a pipeline for EEG channel subset selection that incorporates not only data‐driven methods but also practical considerations such as usability and clinical applicability. This mixed‐methods approach reflects real‐world constraints and aims to support scalable and interpretable seizure detection in resource‐constrained environments.

In this study, we focused on unipolar configurations, as they allow easier attribution of signals to individual electrodes and are more compatible with point‐of‐care EEG devices. To further evaluate the adaptability of the proposed pipeline for wearable EEG applications, future work could explore the use of bipolar configurations. This could be achieved by re‐referencing existing unipolar recordings or testing the pipeline on datasets that natively include bipolar setups [53]. Such an investigation would provide valuable insight into the feasibility of deploying seizure detection models on wearable EEG devices using bipolar montages. Thus, this remains a limitation of the present study and an important direction for future research.

## 5 Conclusions

Our study presents a data‐driven pipeline for EEG channel selection that integrates both performance and usability considerations in EEG‐based classification tasks. Using seizure detection as a representative application, we demonstrate how standard hospital‐collected multichannel EEG recordings can be leveraged to evaluate low‐density montage configurations. Our findings also highlight the consistent presence of temporal and central electrodes in top‐performing configurations, underscoring their importance for robust seizure detection across diverse patient populations. Rather than maximising detection performance alone, the pipeline prioritises clinical constraints and interpretability, offering a practical and scalable approach for real‐world deployment in point‐of‐care EEG systems.

## Author Contributions

**Shima Abdullateef**: formal analysis, methodology, visualisation, writing – original draft. **Iris Soare‐Nguyen**: formal analysis, methodology, visualisation, writing – review and editing. **Brian Jordan**: data curation, writing – review and editing. **Ailsa McLellan**: data curation, writing – review and editing. **Vera Nenadovic**: conceptualisation, writing – review and editing. **Javier Escudero**: conceptualisation, methodology, supervision, writing – review and editing. **Tsz‐Yan Milly Lo**: conceptualisation, supervision, writing – review and editing.

## Funding

The authors gratefully acknowledge support from the Medical Research Council Confidence in Concept scheme and the Chief Scientist Office (Grant code: TCS/21/30). Javier Escudero also acknowledges funding from the Engineering and Physical Sciences Research Council (EPSRC) (Grant number: UKRI1659).

## Conflicts of Interest

Vera Nenadovic holds licensed technology through BrainsView Inc. related to seizure detection software and may benefit financially if the software is commercialised. All other authors declare no conflicts of interest.

## Data Availability

The Siena scalp EEG dataset is publicly available via PhysioNet: https://physionet.org/content/siena‐scalp‐eeg/1.0.0/. The local paediatric dataset may be made available for research purposes upon reasonable request and subject to appropriate ethical approvals and data‐sharing agreements.

## 1

**TABLE A1:** EEG montage combinations identified in Step 0 for their clinical relevance.

C3 C4	T3 T4	P3 P4	O1 O2
F3 C3 C4 F4	T3 P3 P4 T4	F3 P3 P4 F4	P3 FP1 FP2 P4
F7 C3 C4 F8	T3 T5 T6 T4	F7 P3 P4 F8	T3 O1 O2 T4
FP1 C3 C4 FP2	T3 F7 F8 T4	T5 P3 P4 T6	F7 O1 O2 F8
T5 C3 C4 T6	T3 F3 F4 T4	P3 C3 C4 P4	F3 O1 O2 F4
T3 C3 C4 T4	T3 FP1 FP2 T4	P3 O1 O2 P4	FP1 O1 O2 FP2
C3 O1 O2 C4			
